# Predicting Early Mortality in Adult Trauma Patients Admitted to Three Public University Hospitals in Urban India: A Prospective Multicentre Cohort Study

**DOI:** 10.1371/journal.pone.0105606

**Published:** 2014-09-02

**Authors:** Martin Gerdin, Nobhojit Roy, Monty Khajanchi, Vineet Kumar, Satish Dharap, Li Felländer-Tsai, Max Petzold, Sanjeev Bhoi, Makhan Lal Saha, Johan von Schreeb

**Affiliations:** 1 Health Systems and Policy, Department of Public Health Sciences, Karolinska Institutet, Stockholm, Sweden; 2 Department of Surgery, Bhabha Atomic Research Centre Hospital, Mumbai, India; 3 School of Habitat, Tata Institute of Social Sciences, Mumbai, India; 4 Department of Surgery, King Edward Memorial Hospital, Mumbai, India; 5 Department of Surgery, Lokmanya Tilak Municipal Medical College and General Hospital, Mumbai, India; 6 Division of Orthopedics and Biotechnology, Department of Clinical Science Intervention and Technology, Karolinska Institutet, Stockholm, Sweden; 7 Centre for Applied Biostatistics, Occupational and Environmental Medicine, Sahlgrenska Academy at University of Gothenburg, Gothenburg, Sweden; 8 Department of Emergency Medicine, Jai Prakash Narayan Apex Trauma Center, All India Institute of Medical Sciences, New Delhi, India; 9 Department of Surgery, Institute of Post-Graduate Medical Education and Research and Seth Sukhlal Karnani Memorial Hospital, Kolkata, India; Azienda Ospedaliero-Universitaria Careggi, Italy

## Abstract

**Background:**

In India alone, more than one million people die yearly due to trauma. Identification of patients at risk of early mortality is crucial to guide clinical management and explain prognosis. Prediction models can support clinical judgement, but existing models have methodological limitations. The aim of this study was to derive a vital sign based prediction model for early mortality among adult trauma patients admitted to three public university hospitals in urban India.

**Methods:**

We conducted a prospective cohort study of adult trauma patients admitted to three urban university hospitals in India between October 2013 and January 2014. The outcome measure was mortality within 24 hours. We used logistic regression with restricted cubic splines to derive our model. We assessed model performance in terms of discrimination, calibration, and optimism.

**Results:**

A total of 1629 patients were included. Median age was 35, 80% were males. Mortality between admission and 24 hours was 6%. Our final model included systolic blood pressure, heart rate, and Glasgow coma scale. Our model displayed good discrimination, with an area under the receiver operating characteristics curve (AUROCC) of 0.85. Predicted mortality corresponded well with observed mortality, indicating good calibration.

**Conclusion:**

This study showed that routinely recorded systolic blood pressure, heart rate, and Glasgow coma scale predicted early hospital mortality in trauma patients admitted to three public university hospitals in urban India. Our model needs to be externally validated before it can be applied in the clinical setting.

## Background

Trauma accounts for more than 10% of the global burden of disease,[1] and every year more people die from trauma than from malaria, tuberculosis, HIV/AIDS, and maternal conditions combined [2]. Of the more than five million annual trauma deaths, over 90% occur in low and middle-income countries [3]. In addition to preventive and pre-hospital measures, efforts are needed to improve hospital trauma care, as between 30 to 50% of all trauma deaths globally occurs in hospitals [4], [5].

India, a lower middle-income country, accounts for almost 20% of global trauma mortality [6]. A significant proportion of India's trauma mortality occurs in urban areas [7], where pre-hospital care is limited [8], [9]. About 50% of India's trauma mortality has been reported to occur in hospitals [10], potentially due to poorly developed emergency care and limited critical care capabilities [11], [12]. Also, many hospitals lack formalised triage systems to rapidly identify patients at risk of mortality [13].

Several clinical prediction models have been developed to be part of such systems and support clinical judgement in identifying trauma patients at risk of mortality, guide management, and explain prognosis [14]–[21]. A systematic review of trauma prediction models found that most existing models had methodological limitations [14]. Also, most trauma mortality prediction models have been based only on data from high-income countries [14]–[17], [21]. Unfortunately, several prediction models include respiratory rate, despite the fact that this vital sign is rarely completely recorded [22]. To improve usefulness, there is a need for models without respiratory rate [23], [24].

Vital signs-based prediction models can easily be used immediately when patients arrive to hospital or already in pre-hospital triage. Hence, such models would be ideal for busy public hospitals in urban India, where patients have not been triaged prior to arrival, and there is lack of resources. We have recently shown that early trauma mortality is a problem in the Indian hospital setting [25], indicating the need for urgent intervention. Therefore, our aim was to derive a vital signs-based prediction model for early mortality among adult trauma patients admitted to three public university hospitals in urban India.

## Methods

### Study design

We conducted a prospective multi-centre observational cohort study in three public university hospitals across India between October 2013 and January 2014. We report our study according to recently published guidelines for prediction research [26].

### Setting

Three centres across India participated in this study. Jai Prakash Narayan Apex Trauma Center (JPNATC), All India Institute of Medical Sciences, New Delhi, is a dedicated trauma centre with almost 180 beds. Lokmanya Tilak Municipal General Hospital (LTMGH), Mumbai is a tertiary level public university hospital with a dedicated trauma ward with 14 beds. The Institute of Post-Graduate Medical Education and Research and Seth Sukhlal Karnani Memorial Hospital (IPGMER & SSKM), Kolkata is also a tertiary level public university hospital, but has no dedicated trauma ward. Instead all emergencies are seen in the same area. We selected these hospitals because the wide range in level of trauma care. The cost of care in all hospitals is nominal and the patients admitted represent mainly a lower socioeconomic stratum of the population.

### Eligibility criteria

Patients aged ≥15 years were included if they presented with history of trauma and were admitted or died between arrival and admission. Patients with isolated limb injury and patients who were dead on arrival were not included.

### Variables

Our outcome measure was early mortality, defined as death within 24 hours of the time when the first vital signs were recorded. Based on external information from published literature and feasibility considerations we specified our full model a priori as including the vital signs systolic blood pressure, heart rate, and Glasgow coma scale as potential predictors. To characterise our sample, we also collected data on descriptive variables such as age, sex, transfer status, mode of transport, and mechanism of injury.

### Data

In each hospital, one data collector prospectively gathered data for eight hours per day through direct observation in the emergency room and data extraction from patient files. The data collector worked day and night shifts according to a rotating schedule. For patients admitted outside of the shift, data was retrospectively collected from patient files within days. All data collectors had at least a health science master degree, and were continuously trained and supervised by project management. They did no perform their own recordings, but relied on the measurements performed by the residents on duty. Patients' outcomes were recorded 24 hours after the time when vital signs were first recorded, at discharge or at death in hospital, whichever occurred first. Data was uploaded on a weekly basis and project management had weekly data review meetings.

### Sample size calculation

We used ten events per free parameter to calculate sample size [27], [28]. An event was defined as a patient who died within 24 hours of the time when the first vital signs were recorded. A free parameter was defined as any variable included in the analysis model. To allow for flexible modelling of potential non-linear associations between predictors and mortality we assumed that the most flexible model would include transformations of systolic blood pressure, heart rate, and Glasgow coma scale so that each vital sign was represented by three variables. Therefore, we included the first 90 events and all non-events enrolled during the same time period.

### Statistical methods

We used Stata (Stata: Release 13. StataCorp LP, College Station, Texas) for statistical analyses. Where applicable a confidence level of 95% and a significance level of 5% were used.

#### Missing data strategy

To maximise efficiency and assuming that data was missing at random, we used multiple imputation using chained equations to handle missing data.[29] We explored patterns of missingness and variable distributions before we decided on the imputation model. Variables were imputed using linear regression, predictive mean matching, nominal or ordinal logistic regression as appropriate. As recommended, mortality was included in the imputation model [30]. To estimate the number of imputed dataset that we needed to create we calculated the proportion of patients who had at least one missing value in any of the vital signs or descriptive variables in each hospital [30], [31]. We then created as many datasets as the proportion of incomplete cases in the hospital with most missing data. We first imputed each hospital's dataset separately and then combined all datasets for analysis.

#### Modelling approach

In the primary analysis, a patient who was discharged alive before 24 hours was considered to be alive at 24 hours. We screened univariate associations between vital signs and early mortality using lowess regression and visually assessed departures from linearity on the log odds scale. We used logistic regression with a stepwise backward selection approach to derive our model. Restricted cubic splines were used to flexible model potential non-linear associations between vital signs and mortality log odds [32], [33]. Knots were placed at equally spaced percentiles [33]. The selection algorithm terminated when all included variables were significant at the 0.2 level. Estimated coefficients and variances across imputed datasets were combined using Rubin's rules [29], [34]. The complete modelling procedure was replicated in 300 separately imputed bootstrap samples drawn with replacement. The final model included only variables that were selected in the original sample and >50% of bootstrap samples.

#### Model performance and internal validation

We assessed performance of our final model in terms of discrimination, calibration, and optimism [35]. To assess discrimination, we calculated the area under the receiver operating characteristics curve (AUROCC). To assess calibration, we plotted the proportion of patients with early mortality across quantiles of predicted probability of mortality, calculated the calibration slope, and used the Hosmer-Lemeshow test. We report discrimination and calibration measures using their median and interquartile range (IQR) across imputed datasets [36], [37]. To estimate optimism we used the same bootstrap procedure as above. For each bootstrap model the AUROCC was estimated in both the bootstrap sample and the original sample. We then estimated optimism as the mean difference in AUROCC between the bootstrap and the original samples. Furthermore, we used the bootstrap procedure to estimate a linear shrinkage factor that was applied to the final model's coefficients to limit potential overfitting [38].

#### Sensitivity analyses

First, we compared the performance of our final model with the performance of a “reduced model” that included only the two strongest predictors. Second, we compared our final model with a “full model” that included age, sex, transfer status, and mechanism of injury in addition to systolic blood pressure, heart rate, and Glasgow coma scale. To maximise discrimination of this full model additional variables were included regardless of their statistical significance. Third, we assessed the performance of our final model under the worst-case-scenario that all patients discharged alive before 24 hours were dead at 24 hours. Finally, we performed a complete case analysis in the original sample and compared with the analysis based on imputed data.

### Ethical considerations

Ethical clearance was obtained from each of the participating hospital. The names of the ethical bodies and reference numbers were Institute Ethics Committee All India Institute of Medical Sciences (EC/NP-279/2013 RP-Ol/2013), Ethics Committee of the Staff and Research Society (IEC/11/13), and IPGME&R Research Oversight Committee (IEC/279) for JPNATC, LTMGH, and SSKM respectively. We applied for a waiver of informed consent, which was granted by all review boards. The patients included in this study were all admitted after trauma, often arriving with an altered level of consciousness and in severe physical and psychological distress. As this study involved only collection of routine data, which was later analysed anonymously, and did not alter the care provided in any way, we and the ethics committees felt that obtaining informed consent would be to burden the patients or their relatives unnecessarily.

## Results

A total of 1629 patients were included (Table 1). Out of these, 6% died within 24 hours from the time when vital signs were first recorded. Twenty-nine (2%) patients were discharged alive before 24 hours and hence defined as alive at 24 hours in our primary analysis. Most patients were males (80%) and the median age was 35 (IQR = 24-47) years. Road traffic injury (46%) and falls (27%) were the two most common mechanisms of injury. A majority (68%) of patients were transferred from other facilities. In variables with missing values the fraction of missing data ranged from 6% for time from injury to arrival to the study centres to 20% for systolic blood pressure. Across hospitals the proportion of incomplete cases ranged from 1 to 51%. We therefore created 51 imputed datasets.

**Table 1:** Sample characteristics*.

	Survivors n = 1523	Non-survivors n = 90	Fraction of missing data %
**Age**	34 (24–46)	35 (25–50)	0
**Male (%)**	1241 (81)	66 (73)	0
**Time from injury to arrival (hours)**	8 (3–30)	4 (2–10)	6
**Transferred (%)**	1056 (69)	48 (53)	0
**Mechanism of injury %**			0
Fall	430 (28)	15 (17)	
Railway accident	103 (7)	8 (9)	
Road traffic accident	703 (46)	54 (60)	
Assault	138 (9)	6 (7)	
Burn	95 (6)	4 (4)	
Other	66 (4)	3 (3)	
Unknown	4 (0)	. (.)	
**Systolic blood pressure**	118 (110–125)	94 (80–116)	20
**Heart rate**	88 (80–98)	90 (78–108)	18
**Glasgow coma scale**	15 (10–15)	4 (3–10)	20

*Data is presented as median (IQR) or number (%) as appropriate. Abbreviations: IQR Inter Quartile Range.

**Figure 1:**
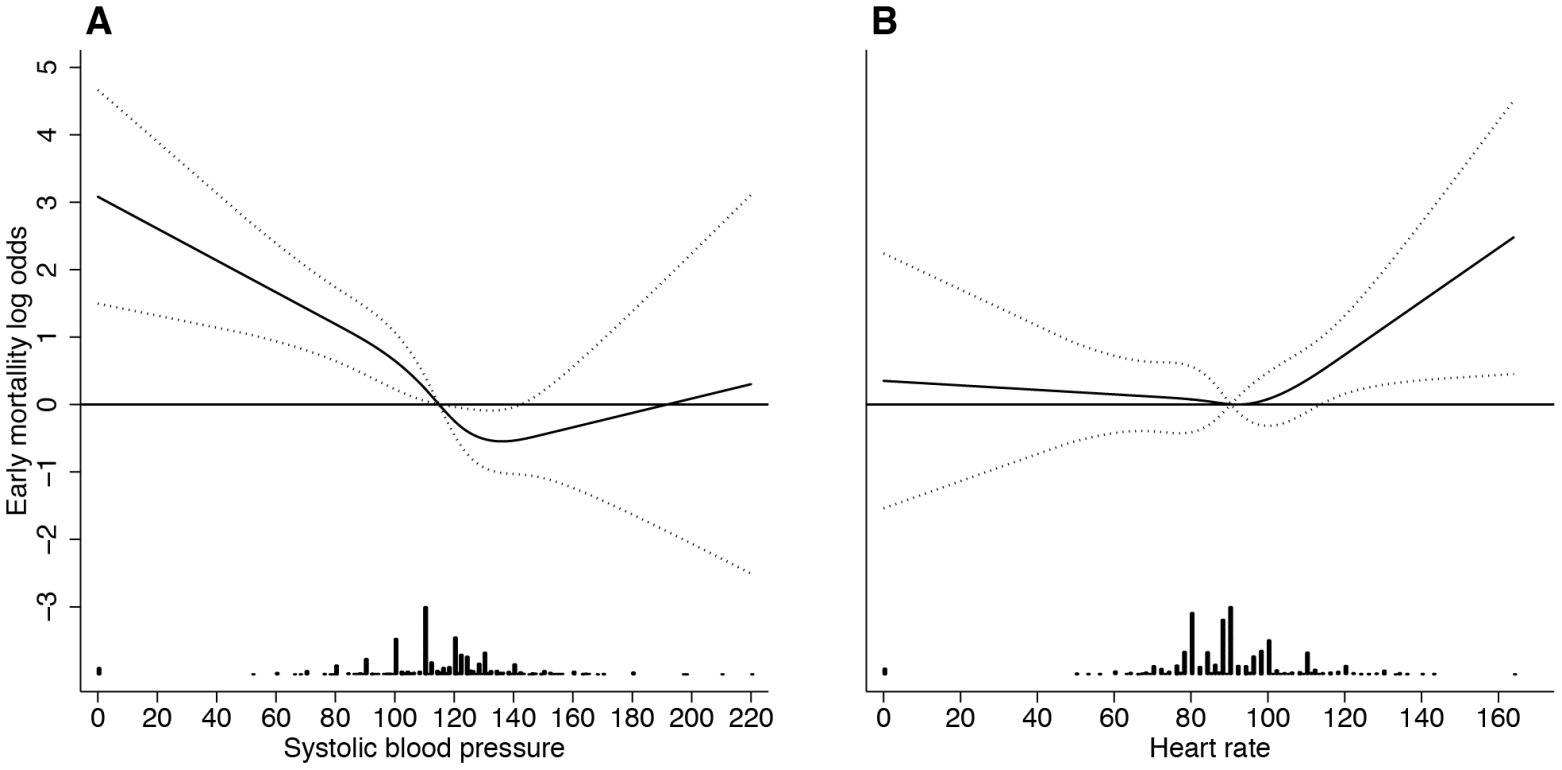
A–B. Nonlinear associations between systolic blood pressure, heart rate, and early mortality modelled using restricted cubic splines. The horizontal solid lines represent log odds = 0. The dotplots in the bottom represent density of observed values. A. Adjusted for heart rate and Glasgow coma scale. B. Adjusted för systolic blood pressure and Glasgow coma scale.

**Figure 2:**
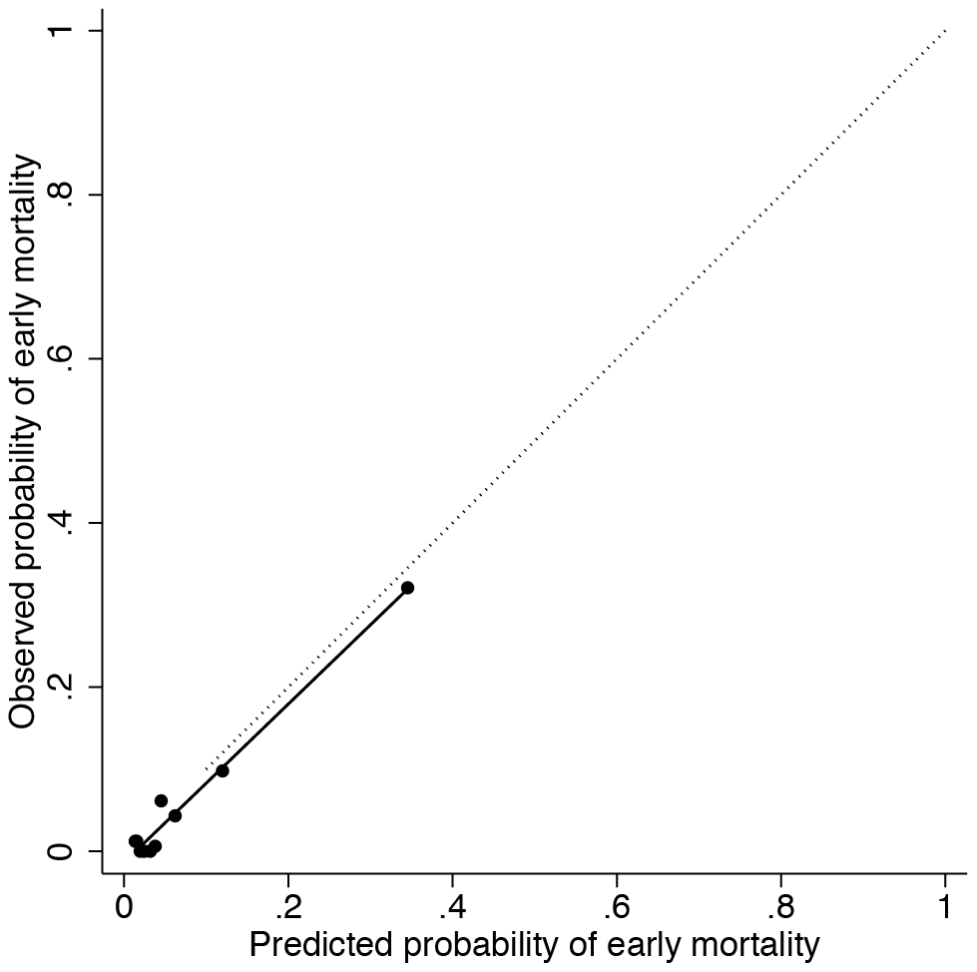
Calibration plot after shrinkage for the risk of early mortality among adult trauma patients model. The dotted line represents perfect calibration. The solid line is the calibration line, calibration slope is 0.96.

Univariate lowess regression indicated that systolic blood pressure and heart rate had a non-linear association with mortality, whereas Glasgow coma scale displayed a linear association. In multivariable analysis the stepwise algorithm terminated with systolic blood pressure as a restricted cubic spline function with four knots, heart rate as a restricted cubic spline function with four knots and Glasgow coma scale as a linear term (Table 2) (Figure 1). The median discrimination across imputed datasets was 0.85 (IQR 0.85–0.86). The median Hosmer-Lemeshow P-value across imputed datasets was 0.33 (IQR 0.14–0.67), indicating adequate calibration (Figure 2). Specificity at 90 and 95% sensitivity was 55 and 21% respectively (Table 3). Optimism was 0.02, and hence the expected performance reduction in external validation is limited. The linear shrinkage factor was 0.90. Final model are given in appendix 1.

**Table 2:** Final model estimates.

	Before shrinkage		After shrinkage*
	Coefficient (95% CI)	P-value	Coefficient (95% CI)
Systolic blood pressure, spline basis function 1	−0.02 (−0.04—0.01)	<0.01**	−0.02 (−0.04—0.01)
Systolic blood pressure, spline basis function 2	−0.04 (−0.12-0.03)		−0.04 (−0.10-0.03)
Systolic blood pressure, spline basis function 3	0.38 (−0.12-0.88)		0.34 (−0.11-0.79)
Heart rate, spline basis function 1	−0.00 (−0.03-0.02)	0.07**	−0.00 (−0.03-0.02)
Heart rate, spline basis function 2	−0.02 (−0.26-0.22)		−0.02 (−0.23-0.20)
Heart rate, spline basis function 3	0.22 (−0.88-1.33)		0.20 (−0.79-1.19)
Glasgow coma scale	−0.23 (−0.29—0.17)	<0.01	−0.20 (-0.26—0.15)
Constant	2.21 (0.91–3.51)	<0.01	NA

* The shrinkage factor was 0.90. ** The significance of spline functions was tested with a joint test of all spline coefficients belonging to the same vital sign being simultaneously equal to 0. Abbreviations: CI Confidence Interval, NA Not Applicable.

**Table 3:** Discrimination measures at selected sensitivity cut-offs*.

Sensitivity	Specificity	Cut-off, predicted probability	Number of false positives (%)	Number of false negatives (%)
95	21	0.01	1219 (79)	4 (4)
90	55	0.02	697 (45)	9 (10)
85	68	0.03	488 (32)	13 (14)
80	78	0.05	345 (22)	18 (20)
75	84	0.07	248 (16)	22 (24)
70	88	0.09	192 (12)	27 (30)
65	90	0.10	156 (10)	31 (34)
60	92	0.12	128 (8)	36 (40)
55	95	0.16	82 (5)	40 (44)
50	96	0.19	61 (4)	45 (50)

* Specificity, cut-offs, and false positives and negatives are reported as their median across imputed datasets.

**Figure 3:**
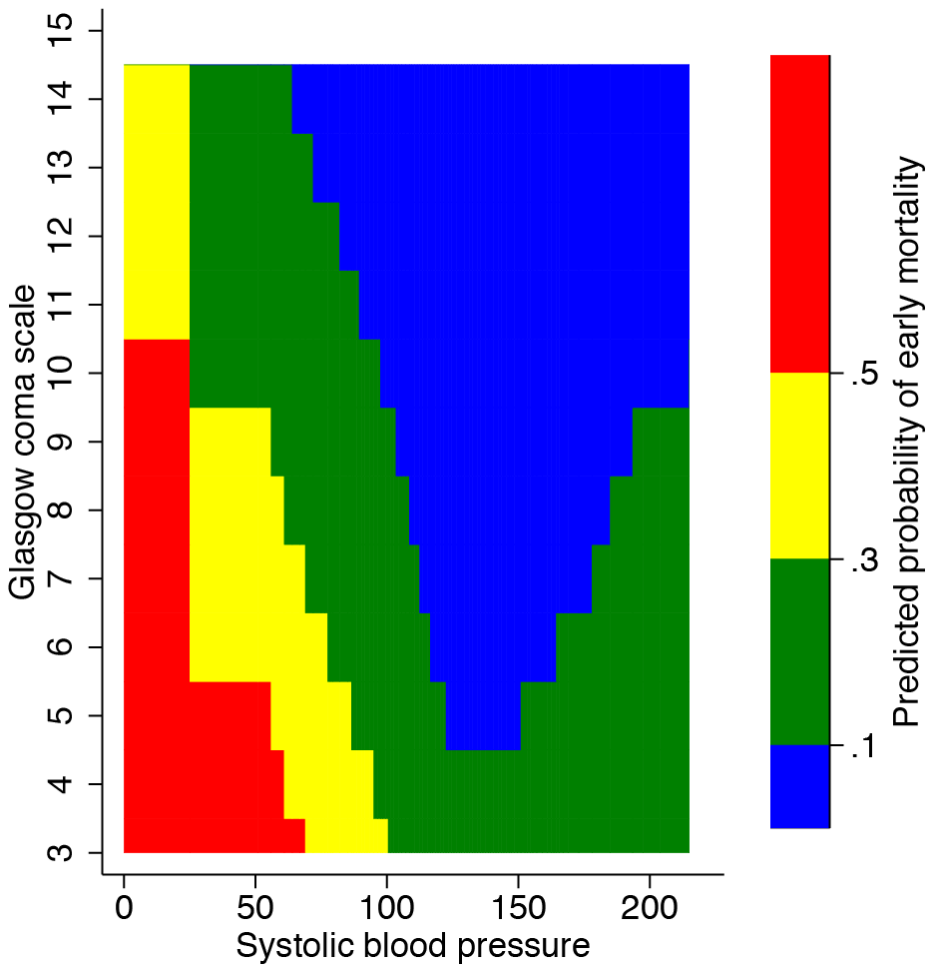
Heatmap based on reduced model with only systolic blood pressure and Glasgow coma scale.

Sensitivity analyses showed that a reduced model with only systolic blood pressure and Glasgow coma scale performed on level with the model that also included heart rate in terms of discrimination (reduced model median AUROCC 0.85, median P-value 0.49) (Figure 3). Furthermore, a full model did not perform significantly better (full model median AUROCC 0.87, median P-value 0.06). Under the worst-case-scenario analysis, in which all patients discharged alive before 24 hours were considered dead at 24 hours, median discrimination was reduced to 0.74 (IQR 0.73–0.75). In complete case analysis systolic blood pressure was included as a spline transformation with four knots, while heart rate was included as a spline transformation with three knots. Glasgow coma scale was included as a linear term. Coefficient sizes remained largely the same. Discrimination was higher, AUROCC 0.90 (95% CI 0.85–0.94). The discrimination of the model based on imputed values when applied only to complete cases was 0.89 (95% CI 0.84–0.94).

## Discussion

In this study routinely collected systolic blood pressure, heart rate, and Glasgow coma scale predicted early mortality among adult trauma patients within 24 hours from when vital signs were first measured. Also, we showed that a reduced model that included only systolic blood pressure and Glasgow coma scale, performed on par with a model that also included heart rate. In terms of potential clinical implications, a model such as our may prove useful in the often chaotic emergency room setting in the studied hospitals. Here, it is often the first year surgical resident on duty who triages patients. Using a prediction tool will help her or him to objectively assess prognosis, even late in the night after many hours of duty, and use this information in communication with senior colleagues and also inform the relatives. Furthermore, the model could help to focus resource management and optimise level of care.

Our models share the advantages of simple vital sign based trauma scores such as the well established and widely used Revised Trauma Score (RTS) [15], and the less well known Physiologic Severity Score (PSS), developed by Husum et al. as a simplified version of RTS [18], Both models include systolic blood pressure, respiratory rate, and level of consciousness. However, neither RTS nor PSS can be used when respiratory rate is missing or not recorded, and hence our models may prove to be feasible alternatives. We have previously highlighted the need for such models [24], as have Kimura et al.[23] Also, our models do not require any blood parameters, for example base deficit, that models such as the Admission base deficit, International normalized ratio, and Glasgow Coma Scale (BIG) score require [39].

Our model performed well in relation to other mortality prediction models developed on trauma data from low- and middle-income countries, such as those published by Perel et al.[20] They aimed to predict in-hospital mortality within 28 days of injury in adult patients with traumatic bleeding and managed to achieve a discrimination of 0.84 to 0.88 using seven predictors; Glasgow coma scale, age, systolic blood pressure, geographical region (low, middle, or high-income country), heart rate, time since injury, and type of injury. However, Perel et al. targeted a specific subgroup of the trauma patient population and hence their results are of unknown applicability to a general trauma population.

### Methodological considerations

First, the inclusion criteria in this study were broad. We argue that broad inclusion criteria result in a more heterogeneous study population that may increase external validity of prediction models. Second, our data is based on routine vital signs recordings. In other words, these measurements have been performed by a large number of residents using different equipment. We therefore want to stress that our results describe associations between routine vital signs recordings and hospital mortality rather than associations between the true underlying physiology and hospital mortality. Third, a linear shrinkage factor was used to improve external validity. Whereas there are other methods, the linear shrinkage factor is perhaps the most straightforward [38]. Finally, the fact that almost 70% of patients were initially treated somewhere else highlights that our results are context specific. However, all three participating hospitals had high transfer rates which indicate that our model may be generalised to this type of setting while it must be studied in detail for less specialised hospitals. Similar transfer rates have been reported from other university hospitals in the region [40].

### Conclusions

Our results indicate that routinely measured systolic blood pressure, heart rate, and Glasgow coma scale can be used to predict early hospital mortality in patients admitted to public university hospitals in the three sites studied across urban India. Because our models do not include respiratory rate, they may prove to be feasible alternatives to established models. However, our model needs to be externally validated before it can be implemented in the clinical setting.

## Appendix 1

The linear predictor *Y* and predicted probability *F(Y)* are given by the general logit formulas
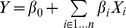
, and 
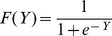
, where β_0_ is the model intercept and β_i_ is the coefficient of covariate X_i_. To explore the fit of our model in your data, you need to create restricted cubic spline basis functions for systolic blood pressure and heart rate, whereas you keep Glasgow coma scale as a linear term. For practical implementation, we used Stata's mkspline program. Using the cubic option, this program by default creates splines with knots located at equally spaced percentiles, as suggested by Harrell.[33] A cubic spline with *n* knots that is restricted to be linear before the first and after the last knot has *n*-1 basis functions, each represented by a new variable. The first basis function *B*_1_ is equal to the original variable. The value for each observation of the subsequent basis functions 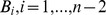, with knot locations 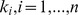, is given by: 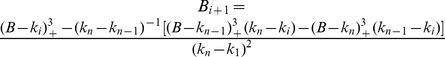 where
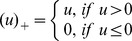
_._[41] In our final model, four knots were used for both systolic blood pressure and heart rate, meaning that knots were located at the 5^th^, 35^th^, 65^th^, and 95^th^ percentiles (Table 4). Now, let *Y*_model_ be the linear predictor from our model. Let *sbp*_1_, *sbp*_2_, and *sbp*_3_ denote the three basis functions for systolic blood pressure, *hr*_1_, *hr*_2_, and *hr*_3_ denote the basis functions for heart rate, and *gcs* denote the linear Glasgow coma scale variable. Using the shrunk coefficients we get:
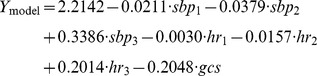



**Table 4:** Knot positions for systolic blood pressure and heart rate at specified percentiles*.

	5^th^ percentile	35^th^ percentile	65^th^ percentile	95^th^ percentile
Systolic blood pressure	80 (80)	110 (110)	121 (122)	147 (149)
Heart rate	70 (70)	86 (85)	92 (92)	118 (119)

*Locations provided before brackets are median values across imputed datasets and within brackets values based on complete case analysis.

The predicted probability of early mortality is calculated using *F(Y*_model_ *)*. We have included a sample do-file as supporting information (File S1) to allow you to explore the fit of our preliminary model in your own data.

## Supporting Information

File S1 **Stata do-file.** This file allows you to explore the fit of our preliminary model in your own dataset using Stata.
(DO) Click here for additional data file.

File S2 **Raw data.** This file includes the data used for this study. Individual hospitals have been masked and are not identifiable.
(CSV)
