# Correction: Predicting Early Mortality in Adult Trauma Patients Admitted to Three Public University Hospitals in Urban India: A Prospective Multicentre Cohort Study

**DOI:** 10.1371/journal.pone.0144886

**Published:** 2015-12-16

**Authors:** Martin Gerdin, Nobhojit Roy, Monty Khajanchi, Vineet Kumar, Satish Dharap, Li Felländer-Tsai, Max Petzold, Sanjeev Bhoi, Makhan Lal Saha, Johan von Schreeb

There is an error in Table 1. The title of the second colum contains the incorrect value. Please see the corrected Table 1 here.

**Table 1:** Sample characteristics*.

	Survivors n = 1539	Non-survivors n = 90	Fraction of missing data %
**Age**	34 (24–46)	35 (25–50)	0
**Male (%)**	1241 (81)	66 (73)	0
**Time from injury to arrival (hours)**	8 (3–30)	4 (2–10)	6
**Transferred (%)**	1056 (69)	48 (53)	0
**Mechanism of injury %**			0
Fall	430 (28)	15 (17)	
Railway accident	103 (7)	8 (9)	
Road traffic accident	703 (46)	54 (60)	
Assault	138 (9)	6 (7)	
Burn	95 (6)	4 (4)	
Other	66 (4)	3 (3)	
Unknown	4 (0)	. (.)	
**Systolic blood pressure**	118 (110–125)	94 (80–116)	20
**Heart rate**	88 (80–98)	90 (78–108)	18
**Glasgow coma scale**	15 (10–15)	4 (3–10)	20

*Data is presented as median (IQR) or number (%) as appropriate. Abbreviations: IQR Inter Quartile Range
